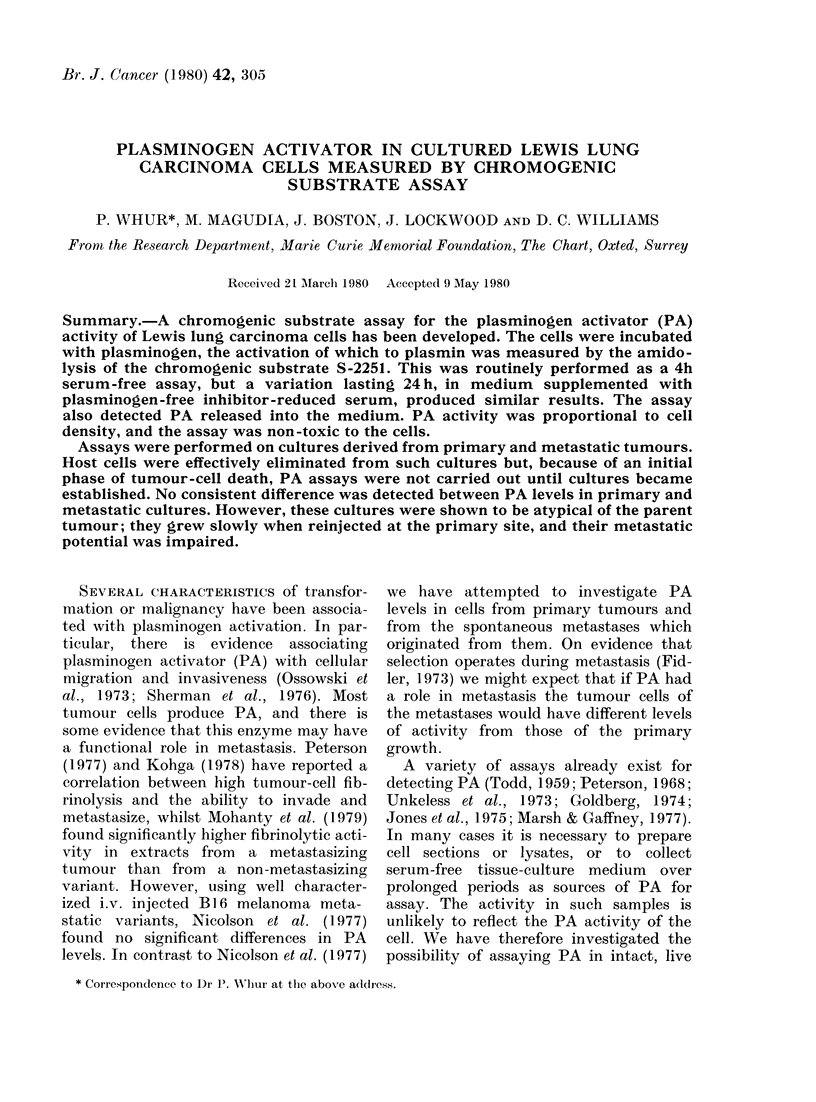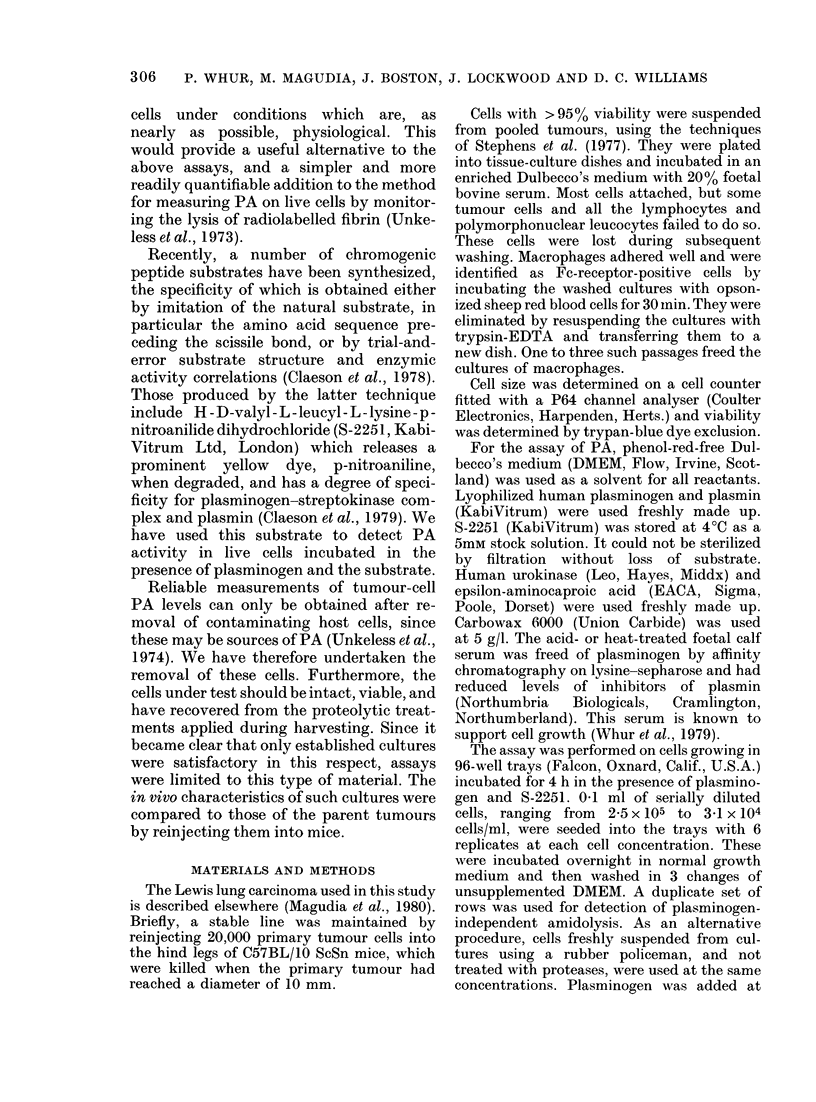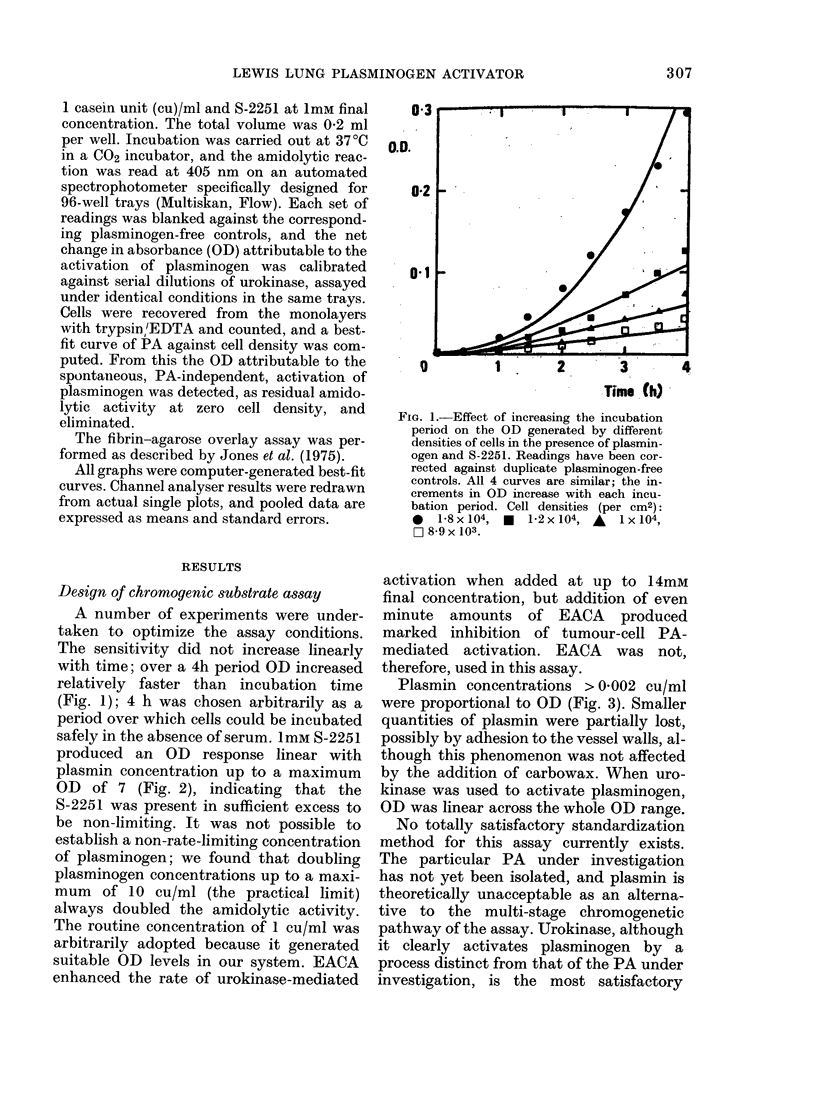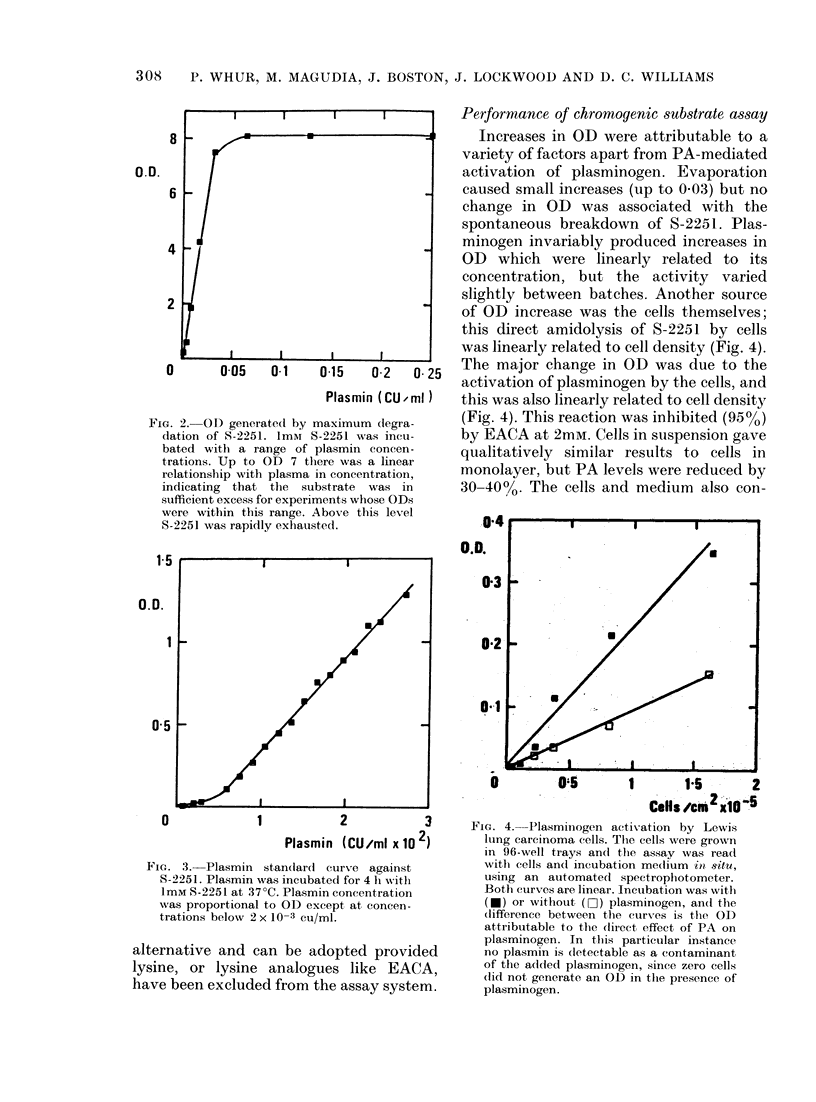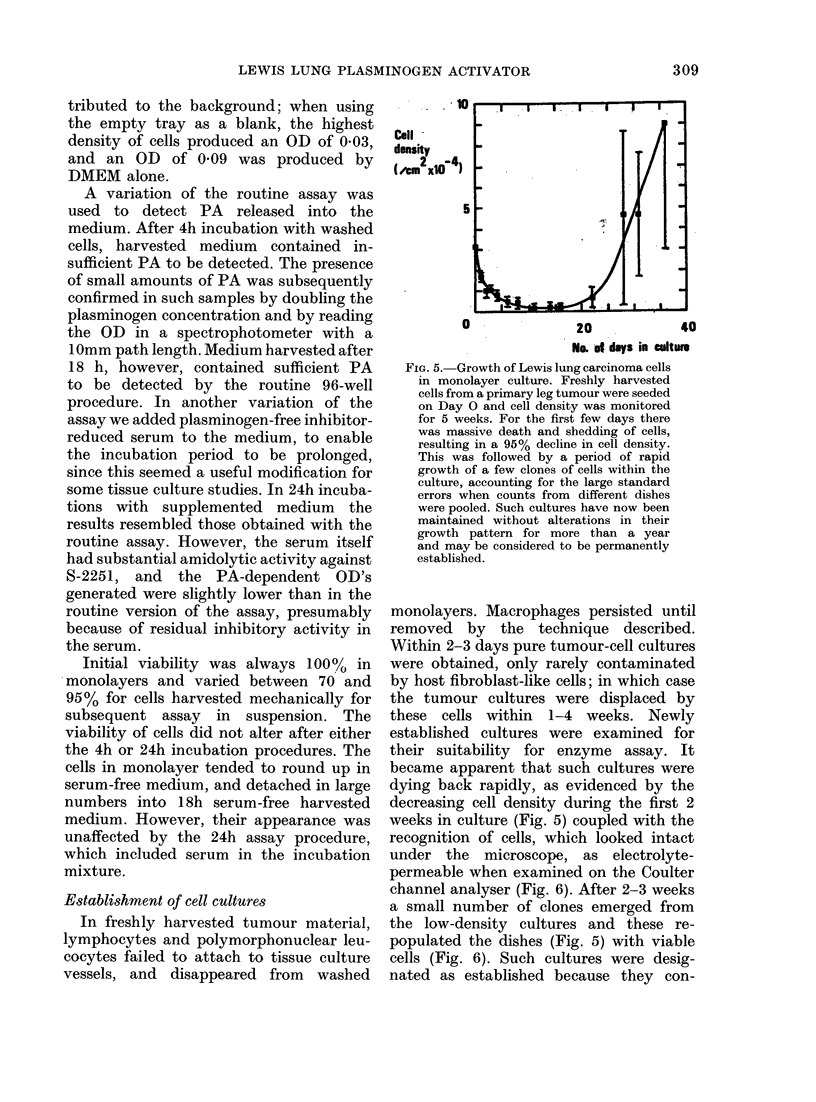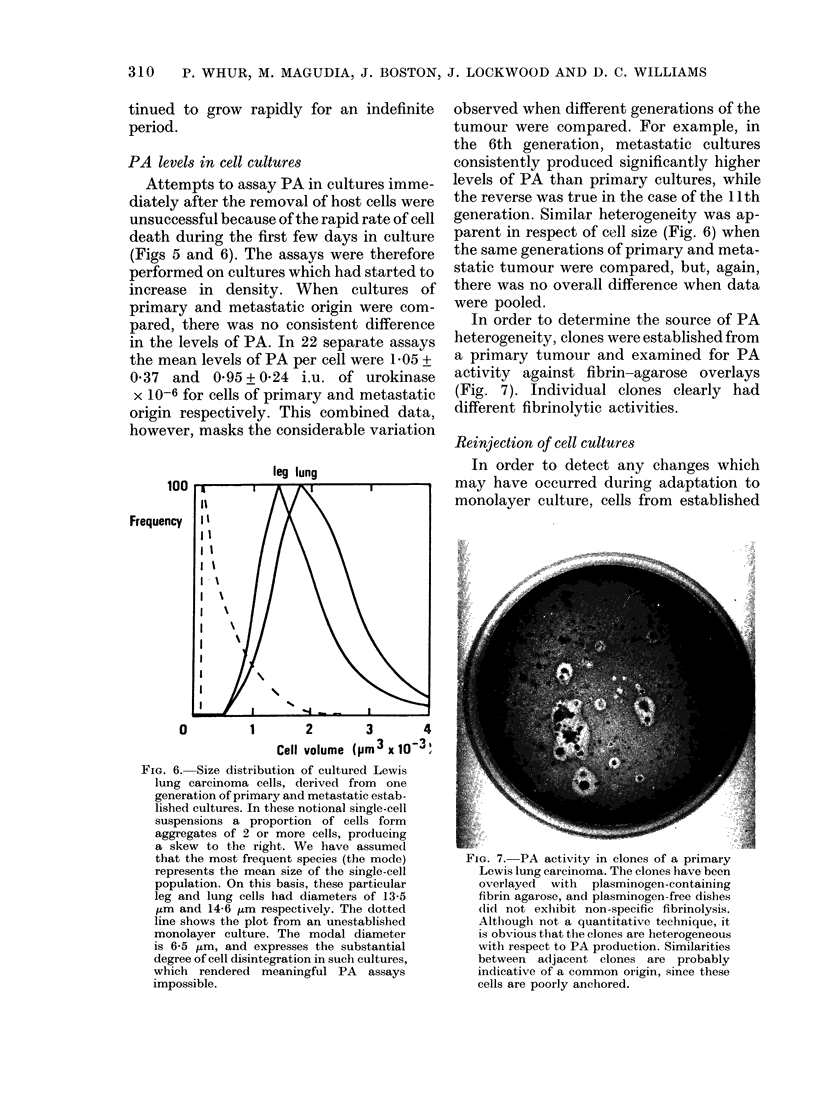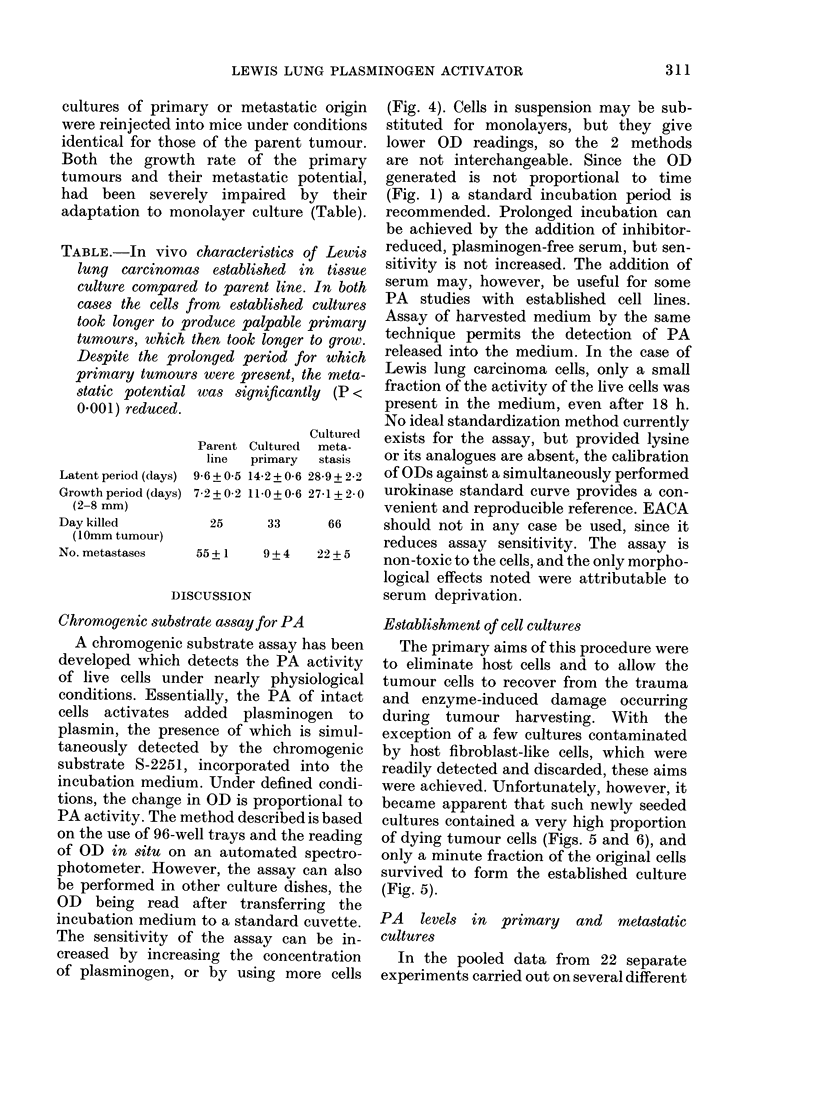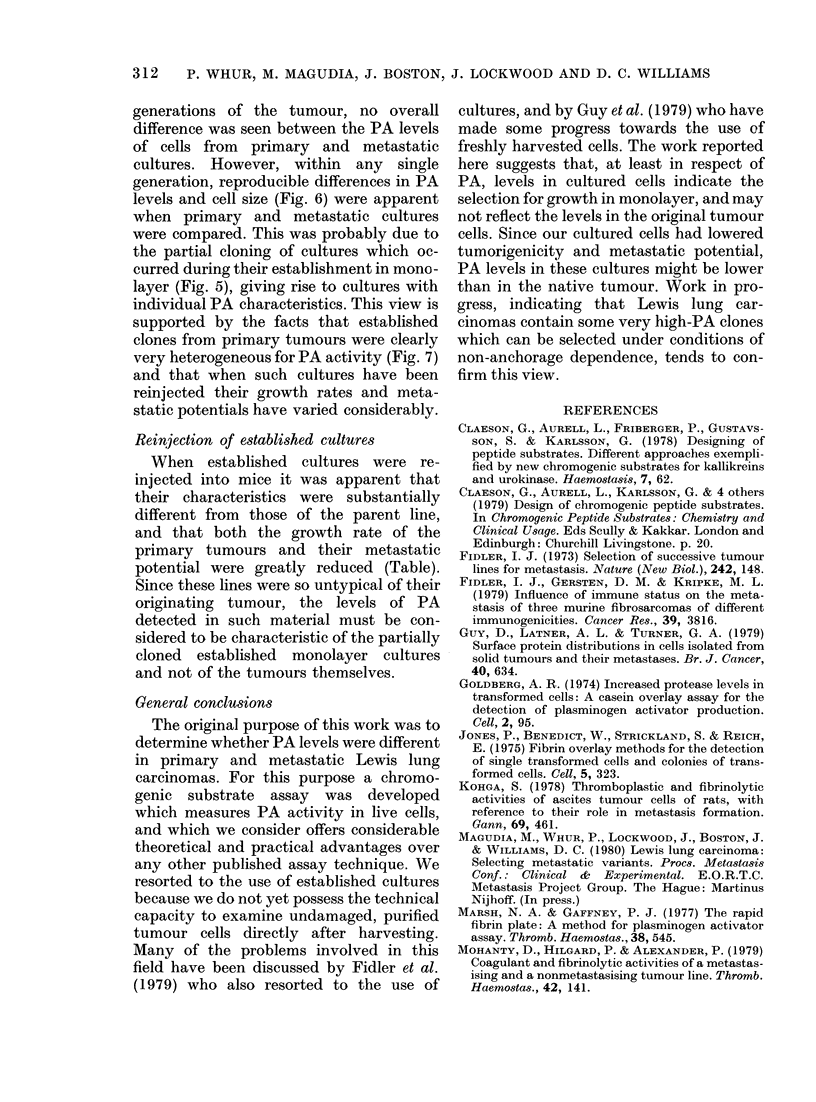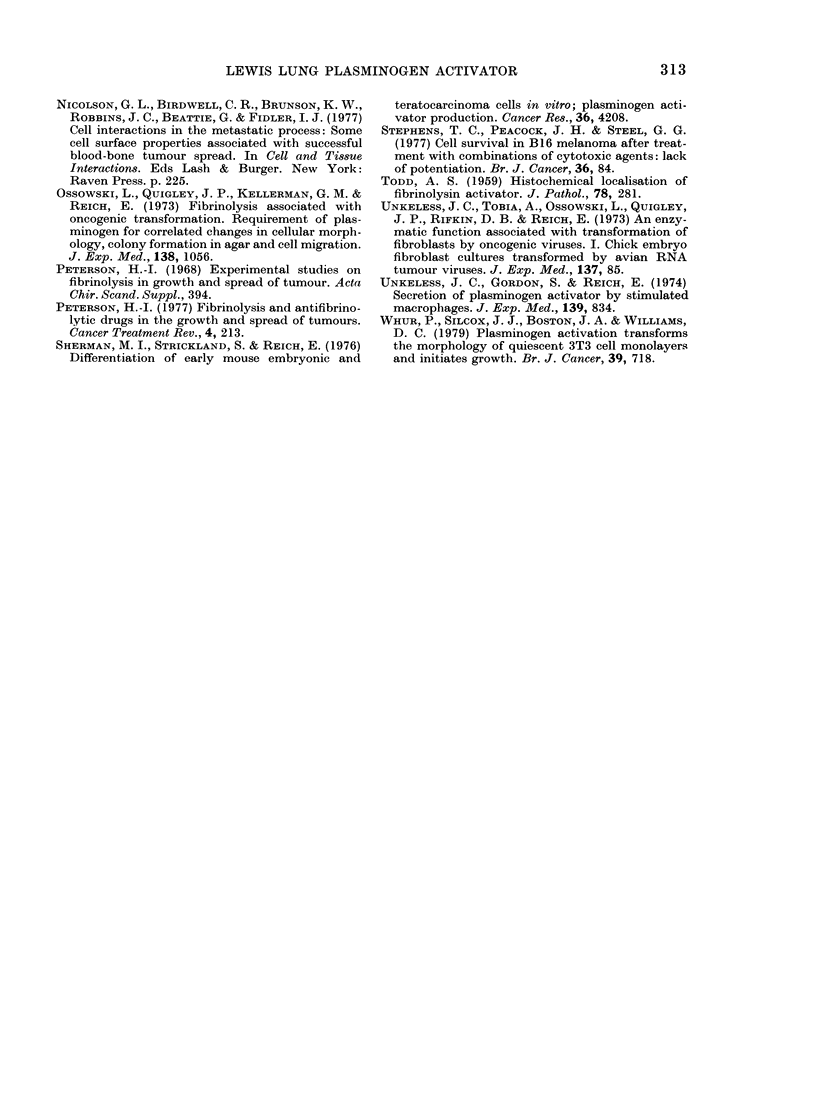# Plasminogen activator in cultured Lewis lung carcinoma cells measured by chromogenic substrate assay.

**DOI:** 10.1038/bjc.1980.231

**Published:** 1980-08

**Authors:** P. Whur, M. Magudia, J. Boston, J. Lockwood, D. C. Williams

## Abstract

**Images:**


Br. J. Cancer (1980) 42, 305

### PLASMINOGEN ACTIVATOR IN CULTURED LEWIS LUNG CARCINOMA CELLS MEASURED BY CHROMOGENIC SUBSTRATE ASSAY

P. WHUR*, M. MAGUDIA, J. BOSTON, J. LOCKWOOD AND D. C. WILLIAMS

*From the Research Department, Marie Curie Memorial Foundation, The Chart, Oxted, Surrey*

Received 21 March 1980 Accepted 9 May 1980

**Summary.—A chromogenic substrate assay for the plasminogen activator (PA) activity of Lewis lung carcinoma cells has been developed. The cells were incubated with plasminogen, the activation of which to plasmin was measured by the amidolysis of the chromogenic substrate S-2251. This was routinely performed as a 4h serum-free assay, but a variation lasting 24h, in medium supplemented with plasminogen-free inhibitor-reduced serum, produced similar results. The assay also detected PA released into the medium. PA activity was proportional to cell density, and the assay was non-toxic to the cells.**

**Assays were performed on cultures derived from primary and metastatic tumours. Host cells were effectively eliminated from such cultures but, because of an initial phase of tumour-cell death, PA assays were not carried out until cultures became established. No consistent difference was detected between PA levels in primary and metastatic cultures. However, these cultures were shown to be atypical of the parent tumour; they grew slowly when reinjected at the primary site, and their metastatic potential was impaired.**

SEVERAL CHARACTERISTICS of transformation or malignancy have been associated with plasminogen activation. In particular, there is evidence associating plasminogen activator (PA) with cellular migration and invasiveness (Ossowski *et al.*, 1973; Sherman *et al.*, 1976). Most tumour cells produce PA, and there is some evidence that this enzyme may have a functional role in metastasis. Peterson (1977) and Kohga (1978) have reported a correlation between high tumour-cell fibrinolysis and the ability to invade and metastasize, whilst Mohanty *et al.* (1979) found significantly higher fibrinolytic activity in extracts from a metastasizing tumour than from a non-metastasizing variant. However, using well characterized i.v. injected B16 melanoma metastatic variants, Nicolson *et al.* (1977) found no significant differences in PA levels. In contrast to Nicolson *et al.* (1977) we have attempted to investigate PA levels in cells from primary tumours and from the spontaneous metastases which originated from them. On evidence that selection operates during metastasis (Fidler, 1973) we might expect that if PA had a role in metastasis the tumour cells of the metastases would have different levels of activity from those of the primary growth.

A variety of assays already exist for detecting PA (Todd, 1959; Peterson, 1968; Unkeless *et al.*, 1973; Goldberg, 1974; Jones *et al.*, 1975; Marsh & Gaffney, 1977). In many cases it is necessary to prepare cell sections or lysates, or to collect serum-free tissue-culture medium over prolonged periods as sources of PA for assay. The activity in such samples is unlikely to reflect the PA activity of the cell. We have therefore investigated the possibility of assaying PA in intact, live

* Correspondence to Dr P. Whur at the above address.

cells under conditions which are, as nearly as possible, physiological. This would provide a useful alternative to the above assays, and a simpler and more readily quantifiable addition to the method for measuring PA on live cells by monitoring the lysis of radiolabelled fibrin (Unkeless *et al.*, 1973).

Recently, a number of chromogenic peptide substrates have been synthesized, the specificity of which is obtained either by imitation of the natural substrate, in particular the amino acid sequence preceding the scissile bond, or by trial-and-error substrate structure and enzymic activity correlations (Claeson *et al.*, 1978). Those produced by the latter technique include H-D-valyl-L-leucyl-L-lysine-p-nitroanilide dihydrochloride (S-2251, KabiVitrum Ltd, London) which releases a prominent yellow dye, p-nitroaniline, when degraded, and has a degree of specificity for plasminogen–streptokinase complex and plasmin (Claeson *et al.*, 1979). We have used this substrate to detect PA activity in live cells incubated in the presence of plasminogen and the substrate.

Reliable measurements of tumour-cell PA levels can only be obtained after removal of contaminating host cells, since these may be sources of PA (Unkeless *et al.*, 1974). We have therefore undertaken the removal of these cells. Furthermore, the cells under test should be intact, viable, and have recovered from the proteolytic treatments applied during harvesting. Since it became clear that only established cultures were satisfactory in this respect, assays were limited to this type of material. The *in vivo* characteristics of such cultures were compared to those of the parent tumours by reinjecting them into mice.

### MATERIALS AND METHODS

The Lewis lung carcinoma used in this study is described elsewhere (Magudia *et al.*, 1980). Briefly, a stable line was maintained by reinjecting 20,000 primary tumour cells into the hind legs of C57BL/10 ScSn mice, which were killed when the primary tumour had reached a diameter of 10 mm.

Cells with $>95\%$ viability were suspended from pooled tumours, using the techniques of Stephens *et al.* (1977). They were plated into tissue-culture dishes and incubated in an enriched Dulbecco's medium with 20% foetal bovine serum. Most cells attached, but some tumour cells and all the lymphocytes and polymorphonuclear leucocytes failed to do so. These cells were lost during subsequent washing. Macrophages adhered well and were identified as Fc-receptor-positive cells by incubating the washed cultures with opsonized sheep red blood cells for 30 min. They were eliminated by resuspending the cultures with trypsin-EDTA and transferring them to a new dish. One to three such passages freed the cultures of macrophages.

Cell size was determined on a cell counter fitted with a P64 channel analyser (Coulter Electronics, Harpenden, Herts.) and viability was determined by trypan-blue dye exclusion.

For the assay of PA, phenol-red-free Dulbecco's medium (DMEM, Flow, Irvine, Scotland) was used as a solvent for all reactants. Lyophilized human plasminogen and plasmin (KabiVitrum) were used freshly made up. S-2251 (KabiVitrum) was stored at 4°C as a 5mM stock solution. It could not be sterilized by filtration without loss of substrate. Human urokinase (Leo, Hayes, Middx) and epsilon-aminocaproic acid (EACA, Sigma, Poole, Dorset) were used freshly made up. Carbowax 6000 (Union Carbide) was used at 5 g/l. The acid- or heat-treated foetal calf serum was freed of plasminogen by affinity chromatography on lysine–sepharose and had reduced levels of inhibitors of plasmin (Northumbria Biologicals, Cramlington, Northumberland). This serum is known to support cell growth (Whur *et al.*, 1979).

The assay was performed on cells growing in 96-well trays (Falcon, Oxnard, Calif., U.S.A.) incubated for 4 h in the presence of plasminogen and S-2251. 0·1 ml of serially diluted cells, ranging from $2{\cdot}5\times10^5$ to $3{\cdot}1\times10^4$ cells/ml, were seeded into the trays with 6 replicates at each cell concentration. These were incubated overnight in normal growth medium and then washed in 3 changes of unsupplemented DMEM. A duplicate set of rows was used for detection of plasminogen-independent amidolysis. As an alternative procedure, cells freshly suspended from cultures using a rubber policeman, and not treated with proteases, were used at the same concentrations. Plasminogen was added at

1 casein unit (cu)/ml and S-2251 at 1mM final concentration. The total volume was 0·2 ml per well. Incubation was carried out at 37 °C in a $CO_2$ incubator, and the amidolytic reaction was read at 405 nm on an automated spectrophotometer specifically designed for 96-well trays (Multiskan, Flow). Each set of readings was blanked against the corresponding plasminogen-free controls, and the net change in absorbance (OD) attributable to the activation of plasminogen was calibrated against serial dilutions of urokinase, assayed under identical conditions in the same trays. Cells were recovered from the monolayers with trypsin/EDTA and counted, and a best-fit curve of PA against cell density was computed. From this the OD attributable to the spontaneous, PA-independent, activation of plasminogen was detected, as residual amidolytic activity at zero cell density, and eliminated.

The fibrin–agarose overlay assay was performed as described by Jones *et al.* (1975).

All graphs were computer-generated best-fit curves. Channel analyser results were redrawn from actual single plots, and pooled data are expressed as means and standard errors.

FIG. 1.—Effect of increasing the incubation period on the OD generated by different densities of cells in the presence of plasminogen and S-2251. Readings have been corrected against duplicate plasminogen-free controls. All 4 curves are similar; the increments in OD increase with each incubation period. Cell densities (per cm$^2$): ● $1{\cdot}8 \times 10^4$, ■ $1{\cdot}2 \times 10^4$, ▲ $1 \times 10^4$, □ $8{\cdot}9 \times 10^3$.

RESULTS

*Design of chromogenic substrate assay*

A number of experiments were undertaken to optimize the assay conditions. The sensitivity did not increase linearly with time; over a 4h period OD increased relatively faster than incubation time (Fig. 1); 4 h was chosen arbitrarily as a period over which cells could be incubated safely in the absence of serum. 1mM S-2251 produced an OD response linear with plasmin concentration up to a maximum OD of 7 (Fig. 2), indicating that the S-2251 was present in sufficient excess to be non-limiting. It was not possible to establish a non-rate-limiting concentration of plasminogen; we found that doubling plasminogen concentrations up to a maximum of 10 cu/ml (the practical limit) always doubled the amidolytic activity. The routine concentration of 1 cu/ml was arbitrarily adopted because it generated suitable OD levels in our system. EACA enhanced the rate of urokinase-mediated activation when added at up to 14mM final concentration, but addition of even minute amounts of EACA produced marked inhibition of tumour-cell PA-mediated activation. EACA was not, therefore, used in this assay.

Plasmin concentrations $> 0{\cdot}002$ cu/ml were proportional to OD (Fig. 3). Smaller quantities of plasmin were partially lost, possibly by adhesion to the vessel walls, although this phenomenon was not affected by the addition of carbowax. When urokinase was used to activate plasminogen, OD was linear across the whole OD range.

No totally satisfactory standardization method for this assay currently exists. The particular PA under investigation has not yet been isolated, and plasmin is theoretically unacceptable as an alternative to the multi-stage chromogenetic pathway of the assay. Urokinase, although it clearly activates plasminogen by a process distinct from that of the PA under investigation, is the most satisfactory

FIG. 2.—OD generated by maximum degradation of S-2251. 1mM S-2251 was incubated with a range of plasmin concentrations. Up to OD 7 there was a linear relationship with plasma in concentration, indicating that the substrate was in sufficient excess for experiments whose ODs were within this range. Above this level S-2251 was rapidly exhausted.

FIG. 3.—Plasmin standard curve against S-2251. Plasmin was incubated for 4 h with 1mM S-2251 at 37°C. Plasmin concentration was proportional to OD except at concentrations below $2 \times 10^{-3}$ cu/ml.

alternative and can be adopted provided lysine, or lysine analogues like EACA, have been excluded from the assay system.

*Performance of chromogenic substrate assay*

Increases in OD were attributable to a variety of factors apart from PA-mediated activation of plasminogen. Evaporation caused small increases (up to 0·03) but no change in OD was associated with the spontaneous breakdown of S-2251. Plasminogen invariably produced increases in OD which were linearly related to its concentration, but the activity varied slightly between batches. Another source of OD increase was the cells themselves; this direct amidolysis of S-2251 by cells was linearly related to cell density (Fig. 4). The major change in OD was due to the activation of plasminogen by the cells, and this was also linearly related to cell density (Fig. 4). This reaction was inhibited (95%) by EACA at 2mM. Cells in suspension gave qualitatively similar results to cells in monolayer, but PA levels were reduced by 30–40%. The cells and medium also con-

FIG. 4.—Plasminogen activation by Lewis lung carcinoma cells. The cells were grown in 96-well trays and the assay was read with cells and incubation medium *in situ*, using an automated spectrophotometer. Both curves are linear. Incubation was with (■) or without (□) plasminogen, and the difference between the curves is the OD attributable to the direct effect of PA on plasminogen. In this particular instance no plasmin is detectable as a contaminant of the added plasminogen, since zero cells did not generate an OD in the presence of plasminogen.

tributed to the background; when using the empty tray as a blank, the highest density of cells produced an OD of 0·03, and an OD of 0·09 was produced by DMEM alone.

A variation of the routine assay was used to detect PA released into the medium. After 4h incubation with washed cells, harvested medium contained insufficient PA to be detected. The presence of small amounts of PA was subsequently confirmed in such samples by doubling the plasminogen concentration and by reading the OD in a spectrophotometer with a 10mm path length. Medium harvested after 18 h, however, contained sufficient PA to be detected by the routine 96-well procedure. In another variation of the assay we added plasminogen-free inhibitor-reduced serum to the medium, to enable the incubation period to be prolonged, since this seemed a useful modification for some tissue culture studies. In 24h incubations with supplemented medium the results resembled those obtained with the routine assay. However, the serum itself had substantial amidolytic activity against S-2251, and the PA-dependent OD's generated were slightly lower than in the routine version of the assay, presumably because of residual inhibitory activity in the serum.

Initial viability was always 100% in monolayers and varied between 70 and 95% for cells harvested mechanically for subsequent assay in suspension. The viability of cells did not alter after either the 4h or 24h incubation procedures. The cells in monolayer tended to round up in serum-free medium, and detached in large numbers into 18h serum-free harvested medium. However, their appearance was unaffected by the 24h assay procedure, which included serum in the incubation mixture.

*Establishment of cell cultures*

In freshly harvested tumour material, lymphocytes and polymorphonuclear leucocytes failed to attach to tissue culture vessels, and disappeared from washed

FIG. 5.—Growth of Lewis lung carcinoma cells in monolayer culture. Freshly harvested cells from a primary leg tumour were seeded on Day O and cell density was monitored for 5 weeks. For the first few days there was massive death and shedding of cells, resulting in a 95% decline in cell density. This was followed by a period of rapid growth of a few clones of cells within the culture, accounting for the large standard errors when counts from different dishes were pooled. Such cultures have now been maintained without alterations in their growth pattern for more than a year and may be considered to be permanently established.

monolayers. Macrophages persisted until removed by the technique described. Within 2–3 days pure tumour-cell cultures were obtained, only rarely contaminated by host fibroblast-like cells; in which case the tumour cultures were displaced by these cells within 1–4 weeks. Newly established cultures were examined for their suitability for enzyme assay. It became apparent that such cultures were dying back rapidly, as evidenced by the decreasing cell density during the first 2 weeks in culture (Fig. 5) coupled with the recognition of cells, which looked intact under the microscope, as electrolyte-permeable when examined on the Coulter channel analyser (Fig. 6). After 2–3 weeks a small number of clones emerged from the low-density cultures and these repopulated the dishes (Fig. 5) with viable cells (Fig. 6). Such cultures were designated as established because they continued to grow rapidly for an indefinite period.

*PA levels in cell cultures*

Attempts to assay PA in cultures immediately after the removal of host cells were unsuccessful because of the rapid rate of cell death during the first few days in culture (Figs 5 and 6). The assays were therefore performed on cultures which had started to increase in density. When cultures of primary and metastatic origin were compared, there was no consistent difference in the levels of PA. In 22 separate assays the mean levels of PA per cell were $1{\cdot}05 \pm 0{\cdot}37$ and $0{\cdot}95 \pm 0{\cdot}24$ i.u. of urokinase $\times 10^{-6}$ for cells of primary and metastatic origin respectively. This combined data, however, masks the considerable variation observed when different generations of the tumour were compared. For example, in the 6th generation, metastatic cultures consistently produced significantly higher levels of PA than primary cultures, while the reverse was true in the case of the 11th generation. Similar heterogeneity was apparent in respect of cell size (Fig. 6) when the same generations of primary and metastatic tumour were compared, but, again, there was no overall difference when data were pooled.

In order to determine the source of PA heterogeneity, clones were established from a primary tumour and examined for PA activity against fibrin–agarose overlays (Fig. 7). Individual clones clearly had different fibrinolytic activities.

*Reinjection of cell cultures*

In order to detect any changes which may have occurred during adaptation to monolayer culture, cells from established

Fig. 6.—Size distribution of cultured Lewis lung carcinoma cells, derived from one generation of primary and metastatic established cultures. In these notional single-cell suspensions a proportion of cells form aggregates of 2 or more cells, producing a skew to the right. We have assumed that the most frequent species (the mode) represents the mean size of the single-cell population. On this basis, these particular leg and lung cells had diameters of 13·5 μm and 14·6 μm respectively. The dotted line shows the plot from an unestablished monolayer culture. The modal diameter is 6·5 μm, and expresses the substantial degree of cell disintegration in such cultures, which rendered meaningful PA assays impossible.

Fig. 7.—PA activity in clones of a primary Lewis lung carcinoma. The clones have been overlayed with plasminogen-containing fibrin agarose, and plasminogen-free dishes did not exhibit non-specific fibrinolysis. Although not a quantitative technique, it is obvious that the clones are heterogeneous with respect to PA production. Similarities between adjacent clones are probably indicative of a common origin, since these cells are poorly anchored.

cultures of primary or metastatic origin were reinjected into mice under conditions identical for those of the parent tumour. Both the growth rate of the primary tumours and their metastatic potential, had been severely impaired by their adaptation to monolayer culture (Table).

TABLE.—In vivo *characteristics of Lewis lung carcinomas established in tissue culture compared to parent line. In both cases the cells from established cultures took longer to produce palpable primary tumours, which then took longer to grow. Despite the prolonged period for which primary tumours were present, the metastatic potential was significantly* ($P < 0.001$) *reduced.*

| | Parent line | Cultured primary | Cultured metastasis |
|---|---|---|---|
| Latent period (days) | $9.6 \pm 0.5$ | $14.2 \pm 0.6$ | $28.9 \pm 2.2$ |
| Growth period (days) (2–8 mm) | $7.2 \pm 0.2$ | $11.0 \pm 0.6$ | $27.1 \pm 2.0$ |
| Day killed (10mm tumour) | 25 | 33 | 66 |
| No. metastases | $55 \pm 1$ | $9 \pm 4$ | $22 \pm 5$ |

### DISCUSSION

### *Chromogenic substrate assay for PA*

A chromogenic substrate assay has been developed which detects the PA activity of live cells under nearly physiological conditions. Essentially, the PA of intact cells activates added plasminogen to plasmin, the presence of which is simultaneously detected by the chromogenic substrate S-2251, incorporated into the incubation medium. Under defined conditions, the change in OD is proportional to PA activity. The method described is based on the use of 96-well trays and the reading of OD *in situ* on an automated spectrophotometer. However, the assay can also be performed in other culture dishes, the OD being read after transferring the incubation medium to a standard cuvette. The sensitivity of the assay can be increased by increasing the concentration of plasminogen, or by using more cells (Fig. 4). Cells in suspension may be substituted for monolayers, but they give lower OD readings, so the 2 methods are not interchangeable. Since the OD generated is not proportional to time (Fig. 1) a standard incubation period is recommended. Prolonged incubation can be achieved by the addition of inhibitor-reduced, plasminogen-free serum, but sensitivity is not increased. The addition of serum may, however, be useful for some PA studies with established cell lines. Assay of harvested medium by the same technique permits the detection of PA released into the medium. In the case of Lewis lung carcinoma cells, only a small fraction of the activity of the live cells was present in the medium, even after 18 h. No ideal standardization method currently exists for the assay, but provided lysine or its analogues are absent, the calibration of ODs against a simultaneously performed urokinase standard curve provides a convenient and reproducible reference. EACA should not in any case be used, since it reduces assay sensitivity. The assay is non-toxic to the cells, and the only morphological effects noted were attributable to serum deprivation.

### *Establishment of cell cultures*

The primary aims of this procedure were to eliminate host cells and to allow the tumour cells to recover from the trauma and enzyme-induced damage occurring during tumour harvesting. With the exception of a few cultures contaminated by host fibroblast-like cells, which were readily detected and discarded, these aims were achieved. Unfortunately, however, it became apparent that such newly seeded cultures contained a very high proportion of dying tumour cells (Figs. 5 and 6), and only a minute fraction of the original cells survived to form the established culture (Fig. 5).

### *PA levels in primary and metastatic cultures*

In the pooled data from 22 separate experiments carried out on several different generations of the tumour, no overall difference was seen between the PA levels of cells from primary and metastatic cultures. However, within any single generation, reproducible differences in PA levels and cell size (Fig. 6) were apparent when primary and metastatic cultures were compared. This was probably due to the partial cloning of cultures which occurred during their establishment in monolayer (Fig. 5), giving rise to cultures with individual PA characteristics. This view is supported by the facts that established clones from primary tumours were clearly very heterogeneous for PA activity (Fig. 7) and that when such cultures have been reinjected their growth rates and metastatic potentials have varied considerably.

*Reinjection of established cultures*

When established cultures were reinjected into mice it was apparent that their characteristics were substantially different from those of the parent line, and that both the growth rate of the primary tumours and their metastatic potential were greatly reduced (Table). Since these lines were so untypical of their originating tumour, the levels of PA detected in such material must be considered to be characteristic of the partially cloned established monolayer cultures and not of the tumours themselves.

*General conclusions*

The original purpose of this work was to determine whether PA levels were different in primary and metastatic Lewis lung carcinomas. For this purpose a chromogenic substrate assay was developed which measures PA activity in live cells, and which we consider offers considerable theoretical and practical advantages over any other published assay technique. We resorted to the use of established cultures because we do not yet possess the technical capacity to examine undamaged, purified tumour cells directly after harvesting. Many of the problems involved in this field have been discussed by Fidler *et al.* (1979) who also resorted to the use of cultures, and by Guy *et al.* (1979) who have made some progress towards the use of freshly harvested cells. The work reported here suggests that, at least in respect of PA, levels in cultured cells indicate the selection for growth in monolayer, and may not reflect the levels in the original tumour cells. Since our cultured cells had lowered tumorigenicity and metastatic potential, PA levels in these cultures might be lower than in the native tumour. Work in progress, indicating that Lewis lung carcinomas contain some very high-PA clones which can be selected under conditions of non-anchorage dependence, tends to confirm this view.

### REFERENCES

CLAESON, G., AURELL, L., FRIBERGER, P., GUSTAVSSON, S. & KARLSSON, G. (1978) Designing of peptide substrates. Different approaches exemplified by new chromogenic substrates for kallikreins and urokinase. *Haemostasis*, **7**, 62.

CLAESON, G., AURELL, L., KARLSSON, G. & 4 others (1979) Design of chromogenic peptide substrates. In *Chromogenic Peptide Substrates: Chemistry and Clinical Usage*. Eds Scully & Kakkar. London and Edinburgh: Churchill Livingstone. p. 20.

FIDLER, I. J. (1973) Selection of successive tumour lines for metastasis. *Nature (New Biol.)*, **242**, 148.

FIDLER, I. J., GERSTEN, D. M. & KRIPKE, M. L. (1979) Influence of immune status on the metastasis of three murine fibrosarcomas of different immunogenicities. *Cancer Res.*, **39**, 3816.

GUY, D., LATNER, A. L. & TURNER, G. A. (1979) Surface protein distributions in cells isolated from solid tumours and their metastases. *Br. J. Cancer*, **40**, 634.

GOLDBERG, A. R. (1974) Increased protease levels in transformed cells: A casein overlay assay for the detection of plasminogen activator production. *Cell*, **2**, 95.

JONES, P., BENEDICT, W., STRICKLAND, S. & REICH, E. (1975) Fibrin overlay methods for the detection of single transformed cells and colonies of transformed cells. *Cell*, **5**, 323.

KOHGA, S. (1978) Thromboplastic and fibrinolytic activities of ascites tumour cells of rats, with reference to their role in metastasis formation. *Gann*, **69**, 461.

MAGUDIA, M., WHUR, P., LOCKWOOD, J., BOSTON, J. & WILLIAMS, D. C. (1980) Lewis lung carcinoma: Selecting metastatic variants. *Procs. Metastasis Conf.: Clinical & Experimental*. E.O.R.T.C. Metastasis Project Group. The Hague: Martinus Nijhoff. (In press.)

MARSH, N. A. & GAFFNEY, P. J. (1977) The rapid fibrin plate: A method for plasminogen activator assay. *Thromb. Haemostas.*, **38**, 545.

MOHANTY, D., HILGARD, P. & ALEXANDER, P. (1979) Coagulant and fibrinolytic activities of a metastasising and a nonmetastasising tumour line. *Thromb. Haemostas.*, **42**, 141.

NICOLSON, G. L., BIRDWELL, C. R., BRUNSON, K. W., ROBBINS, J. C., BEATTIE, G. & FIDLER, I. J. (1977) Cell interactions in the metastatic process: Some cell surface properties associated with successful blood-bone tumour spread. In *Cell and Tissue Interactions*. Eds Lash & Burger. New York: Raven Press. p. 225.

OSSOWSKI, L., QUIGLEY, J. P., KELLERMAN, G. M. & REICH, E. (1973) Fibrinolysis associated with oncogenic transformation. Requirement of plasminogen for correlated changes in cellular morphology, colony formation in agar and cell migration. *J. Exp. Med.*, **138**, 1056.

PETERSON, H.-I. (1968) Experimental studies on fibrinolysis in growth and spread of tumour. *Acta Chir. Scand. Suppl.*, 394.

PETERSON, H.-I. (1977) Fibrinolysis and antifibrinolytic drugs in the growth and spread of tumours. *Cancer Treatment Rev.*, **4**, 213.

SHERMAN, M. I., STRICKLAND, S. & REICH, E. (1976) Differentiation of early mouse embryonic and teratocarcinoma cells *in vitro*; plasminogen activator production. *Cancer Res.*, **36**, 4208.

STEPHENS, T. C., PEACOCK, J. H. & STEEL, G. G. (1977) Cell survival in B16 melanoma after treatment with combinations of cytotoxic agents: lack of potentiation. *Br. J. Cancer*, **36**, 84.

TODD, A. S. (1959) Histochemical localisation of fibrinolysin activator. *J. Pathol.*, **78**, 281.

UNKELESS, J. C., TOBIA, A., OSSOWSKI, L., QUIGLEY, J. P., RIFKIN, D. B. & REICH, E. (1973) An enzymatic function associated with transformation of fibroblasts by oncogenic viruses. I. Chick embryo fibroblast cultures transformed by avian RNA tumour viruses. *J. Exp. Med.*, **137**, 85.

UNKELESS, J. C., GORDON, S. & REICH, E. (1974) Secretion of plasminogen activator by stimulated macrophages. *J. Exp. Med.*, **139**, 834.

WHUR, P., SILCOX, J. J., BOSTON, J. A. & WILLIAMS, D. C. (1979) Plasminogen activation transforms the morphology of quiescent 3T3 cell monolayers and initiates growth. *Br. J. Cancer*, **39**, 718.